# Early changes of muscle membrane properties in porcine faecal peritonitis

**DOI:** 10.1186/s13054-014-0484-2

**Published:** 2014-08-22

**Authors:** Karin A Ackermann, Hugh Bostock, Lukas Brander, Ralph Schröder, Siamak Djafarzadeh, Daniel Tuchscherer, Stephan M Jakob, Jukka Takala, Werner J Z’Graggen

**Affiliations:** Department of Neurology, Inselspital, Bern University Hospital and University of Bern, Freiburgstrasse 4, 3010 Bern, Switzerland; Sobell Department of Motor Neuroscience and Movement Disorders, Institute of Neurology, University College London, Queen Square, London, WC1N 3BG UK; Department of Intensive Care Medicine, Inselspital, Bern University Hospital and University of Bern, Freiburgstrasse 4, 3010 Bern, Switzerland; Departments of Neurology and Neurosurgery, Inselspital, Bern University Hospital and University of Bern, Freiburgstrasse 4, 3010 Bern, Switzerland

## Abstract

**Introduction:**

Sepsis-induced myopathy and critical illness myopathy (CIM) are possible causes of muscle weakness in intensive care patients. They have been attributed to muscle membrane dysfunction. The aim of this study was to investigate membrane properties in the early stage of experimental sepsis by evaluating muscle excitability.

**Methods:**

In total, 20 anaesthetized and mechanically ventilated pigs were randomized to either faecal peritonitis (n = 10) or to non-septic controls (n = 10). Resuscitation with fluids and vasoactive drugs was started 3 hours after peritonitis induction. Muscle membrane properties were investigated by measuring muscle velocity recovery cycles before induction of peritonitis as well as 6, 18 and 27 hours thereafter. Muscle relative refractory period (MRRP) and early supernormality (ESN) were assessed.

**Results:**

Peritonitis lasting 27 hours was associated with an increase of MRRP by 28% from 2.38 ± 0.18 ms (mean ± SD) to 3.47 ± 1.79 ms (*P* <0.01) and a decrease of ESN by 31% from 9.64 ± 2.82% to 6.50 ± 2.64% (*P* <0.01). ESN reduction was already apparent 6 hours after induction of peritonitis. Values in controls did not show any significant alterations.

**Conclusions:**

Muscle membrane abnormalities consistent with membrane depolarization and/or sodium channel inactivation occurred within 6 hours of peritonitis induction. This indicates that changes that have been described in established sepsis-induced myopathy and/or CIM start early in the course of sepsis. Muscle excitability testing facilitates evaluation of the time course of these changes.

**Electronic supplementary material:**

The online version of this article (doi:10.1186/s13054-014-0484-2) contains supplementary material, which is available to authorized users.

## Introduction

Intensive care unit (ICU)-acquired weakness is a frequent and severe complication in ICU patients. Typically, these patients develop diffuse muscle weakness and failure to wean from mechanical ventilation. The entities that variably contribute to ICU-acquired weakness are critical illness polyneuropathy (CIP) and myopathy (CIM) and/or sepsis-induced myopathy [1-3]. Muscle pathologies seem to be much more heterogeneous than CIP and may occur independently of, or in association with, CIP [1-5]. Whereas structural changes have been reported for both [1,2], the pathogenesis of these entities is still a matter of debate. Impaired microcirculation with consecutive bioenergetic failure [1,6], toxic action of cytokines and disturbances in electrolyte gradients [1,3] may all contribute. In addition, the inflammatory response also causes muscle catabolism and loss of muscle function [7-11]. It has also been shown that resting membrane potential is reduced [12].

Muscle fibres are complex electrical organs, in which the accurate control of membrane potential, and the interplay of multiple voltage-dependent ion channels, have essential roles in normal physiological function. Recently, a new protocol for clinical assessment of muscle membranes has been devised. These muscle excitability tests are based on the principle of assessing multi-fibre velocity recovery cycles (VRCs). Using direct muscle stimulation, a column of muscle fibres are excited with paired stimuli separated by a variable inter-stimulus interval. The conduction velocity of the second muscle action potential of each pair changes as a function of the inter-stimulus interval, allowing assessment of the muscle relative refractory period (MRRP) and muscle supernormality [13-19]. These two measurements are sensitive to alterations in muscle membrane potential and ion channel function, and in probable CIM muscle VRC measurements indicate that muscle fibres are depolarized and/or sodium channel inactivation is increased [20].

Up to now, muscle excitability measurements have only been performed in patients diagnosed with probable CIM according to the current guidelines [20]. Considering the likely physiological mechanisms involved, we hypothesized that muscle membrane dysfunction occurs early in sepsis. To test this hypothesis, we studied muscle membrane properties before and during the first 27 hours of experimental sepsis induced by faecal peritonitis in pigs.

## Materials and methods

The study was performed in accordance with the National Institutes of Health guidelines for the care and use of experimental animals and with the approval (No. 100/08) of the ‘Animal Care Committee of the Canton of Bern, Switzerland’. This study was an independent sub-study in a trial of mechanical ventilation. Data on mitochondrial function have been published separately [21].

### Animal preparation

Twenty domestic pigs with an average weight of 40 ± 3 kg (mean ± SD) were fasted overnight and intramuscularly premedicated with ketamine-hydrochloride 100 mg, azaperon 200 mg, and atropine 0.5 mg before induction of anaesthesia and orotracheal intubation. The animals were randomized into a non-septic control group and a faecal peritonitis group (n = 10 in each group), and within these groups to ventilation with either a time-cycled, volume-controlled mode using a tidal volume of 6 to 8 ml/kg, a fraction of inspired oxygen of 0.4, and a positive end-expiratory pressure of 5 cmH_2_O, or using neurally adjusted ventilatory assist (Servo-i™, Maquet Critical Care, Solna, Sweden) (n = 5 per group). In the peritonitis group, 1 g/kg of autologous faeces dissolved in 200 ml glucose 5% was instilled into the abdominal cavity.

Anaesthesia was maintained throughout the experiment with continuous intravenous infusions of ketamine-hydrochloride (200 to 300 mg/h), midazolam (20 to 30 mg/h), and fentanyl (25 to 75 mcg/h). Neuromuscular blocking agents were not used.

Mean arterial blood pressure (MAP) (Pressure Monitoring Set, Edwards Lifesciences LLC, Irvine, CA, USA), cardiac output, and mixed venous oxygen saturation (SvO_2_) (Vigilance II, Edwards Lifesciences LLC, Irvine, CA, USA) were monitored continuously.

A combination of lactated Ringer’s and glucose 50% solutions were administered continuously throughout the experiment at a combined total rate of 5 ml/kg h^−1^. Glucose infusion was adjusted to maintain blood glucose concentration between 3.5 and 6 mmol/l.

Additional fluid boluses and vasoactive drugs were administered according to a haemodynamic protocol [21]. Briefly, a bolus of 50 to 100 ml Ringer’s lactate was administered when MAP was lower than 50 mmHg. The procedure was repeated maximally twice per hour if MAP remained <50 mmHg and if the first bolus had resulted in an increase in stroke volume (measured by intermittent thermodilution). If stroke volume did not increase, a continuous norepinephrine infusion was started and increased to a maximum dose of 600 mcg/h.

### Electrophysiological investigations

The following electrophysiological investigations were performed: standard nerve conduction studies, needle electromyography and muscle excitability measurements. All studies were performed immediately before induction of peritonitis as well as 6, 18 and 27 hours thereafter.

#### Conventional electrophysiological investigations

Motor nerve conduction studies were performed from the ulnar nerve. The nerve was stimulated at the foreleg distal from the elbow. Compound muscle action potentials (CMAPs) were recorded from the interosseus dorsalis I muscle using surface electrodes. Measured parameters were: distal motor latency, CMAP amplitude, duration and stimulus intensity for 50% maximal CMAP amplitude. Concentric needle examinations were performed at multiple insertion points from the extensor digitorum muscle at the foreleg.

#### Muscle excitability measurements

**Figure 1:**
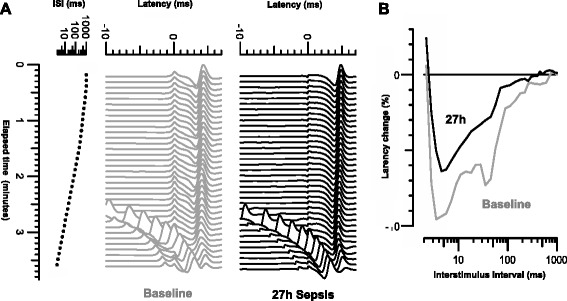
**Recordings of multi-fibre action potentials in pig extensor digitorum at baseline (grey) and 27 hours after induction of sepsis (black). (A)** Recordings of multi-fibre action potentials in pig extensor digitorum at baseline (grey) and 27 hours after induction of sepsis (black). Test stimulus is at latency 0 ms, and conditioning stimuli applied at intervals varied from 1,000 to 1.4 ms. Response latencies were measured to peak after subtraction of response to conditioning stimulus alone. **(B)** Velocity recovery cycles at the two times plotted as percentage changes in latency against the inter-stimulus interval (logarithmic scale).

Multi-fibre responses to direct muscle stimulation were recorded from the foreleg using a recently described protocol [15]. An insulated monopolar needle electrode (TECA, VIASYS Healthcare, MI, Wisconsin, USA) was used as cathode, and a non-polarizable surface electrode as anode. The cathode was inserted into the extensor digitorum muscle and the anode was positioned 15 mm distal to the cathode. For stimulation, rectangular current pulses of 0.05 ms duration were delivered by an isolated linear bipolar current stimulator (DS5, Digitimer Ltd, Welwyn Garden City, UK). Recordings were made with a 30G EMG electrode (Medtronic, Skovlunde, Denmark). The EMG needle was inserted in the muscle about 15 to 25 mm proximal to the cathode. The signal was amplified as described above. Stimulation and recording were controlled by QTRAC software (written by H. Bostock, copyright Institute of Neurology, London, UK). Multi-fibre VRCs with single conditioning stimuli and with test stimuli alone were recorded. Latency changes were measured as conditioning stimuli were applied at inter-stimulus intervals of 2 to 1000 ms (Figure 1). Data analysis has been described in detail in previous articles [15,19]. The following VRC parameters were assessed: (1) muscle relative refractory period in ms (MRRP) and (2) early supernormality (ESN), measured as the peak percentage reduction in latency at inter-stimulus intervals shorter than 15 ms.

### Laboratory examinations

Blood samples including arterial blood gas analysis were taken immediately after the electrophysiological investigations. Sodium, potassium, glucose, lactate, pH, bicarbonate, base excess and tumour necrosis factor alpha (TNFα) were measured. Arterial blood samples were analyzed in a blood gas analyzer (GEM 3000 analyzer, Instrumentation Laboratory, Bedford, MA, USA), for PaO_2_, PaCO_2_ (adjusted to central body temperature), pH, lactate, bicarbonate, base excess (BE), sodium and potassium. For SvO_2_ and haemoglobin, a device calibrated for porcine blood was used (OSM 3, Radiometer, Copenhagen, Denmark). Plasma TNFα was measured using a porcine immunoassay kit (R&D Systems Europe Ltd, Abingdon, UK).

### Muscle morphology

Muscle biopsies were obtained at the end of the experiment from all animals. Cross-sections (12 μm) were cut perpendicular to the longitudinal axis of muscle fibres. Haematoxylin and eosin and myofibrillar ATPase staining was performed. In each animal, a cross-sectional area of skeletal muscle fibres was measured from 50 muscle fibres separately on one slice [22] by using light microscopy and imaging software (NIH Image 1.62, National Institute of Mental Health, Bethesda, MD, USA).

### Densitometric scanning of immune reactive bands of myosin normalized to actin

Densitometric scanning of immune reactive bands of myosin protein normalized to actin was performed at the end of the experiment. Skeletal muscle tissue samples were homogenized in ice-cold lysis buffer (250 mM sucrose, 5 mM MgCl2, 50 mM Tris-HCl, pH 7.4) using a glass pestle. The homogenates were then solubilized in 4X sodium dodecyl sulphate sample buffer containing 100 mM dithiothreitol, centrifuged for 5 min at 16,000 g and the supernatants were collected. The protein concentration of the homogenates (supernatants) was determined with the Quant-iTM assay kit and read with the Qubit-TM fluorometer (Invitrogen, Life Technologies, Zug, Switzerland).

Western blot analysis was performed using the homogenates from all 10 control and 10 peritonitis animals. Equal amounts of protein (10 μg per well) were separated on NuPAGE 4 to 12% gradient gels (Invitrogen, Life Technologies, Zug, Switzerland). Two gels were used (one with 14 and one with 6 wells). Gels were then transferred into nitrocellulose membranes with the iBlot-TM dry blotting system (Invitrogen, Life Technologies, Zug, Switzerland). Equal loading was verified by staining the ex-gels with SimplyBlue™ SafeStain (Invitrogen Life Technologies, Zug, Switzerland). Afterwards, the membrane was blocked for 30 min with the incubation buffer (10 mM Tris-HCl, pH 7.5, 100 mM NaCl and 0.1% w/v Tween 20) supplemented with 5% bovine serum albumin. The membrane was then cut in half and the lower half was incubated with the actin polyclonal antibody (dilution 1:1,000) (Sigma-Aldrich, Buchs, Switzerland), whereas the upper half was incubated with a rabbit skeletal muscle fast myosin heavy chain antibody (Abcam, Cambridge, UK) (dilution 1:1,000) for 2 hours. The membranes were then washed three times with incubation buffer and incubated for 1 hour with horseradish peroxidase goat polyclonal anti-rabbit immunoglobulin (Ig)G (Novus Biologicals, Littleton, CO, USA) (dilution 1:1,000) secondary antibodies. Afterwards, the membranes were developed with the enhanced chemiluminescence detection kit (Thermo Scientific Pierce, Rockford, IL, USA). The membranes were viewed in the ChemiDoc Gel Documentation System (Bio-Rad Laboratories AG, Reinach, Switzerland) for signal detection. Quantification of protein bands (ECL signals) was performed by densitometry using the Quantity One densitometry software (Bio-Rad Laboratories AG, Reinach, Switzerland). The sum of the myosin band pixel intensity (intensity x mm^2^) in each lane was normalized to the corresponding actin intensity (adjusted after local background subtractions for each band) in the same lane.

### Statistical analyses

Statistical analyses were performed using the QTRAC data analysis software (copyright Institute of Neurology, London, UK), which was also used to generate the figures. Because the control and peritonitis groups differed in variance, and because some of the measurements were not normally distributed, differences between the control and peritonitis group for the same time point were tested by applying the Welch test to the ranked data. To compare changes over time within the same treatment group the Friedman test was used. Wilcoxon test was used to compare values at baseline (defined as muscle excitability measurements and blood samples taken immediately before induction of peritonitis) with those at 27 hours. Proportions were compared using the Fisher’s exact test (GraphPad InStat 3.05; GraphPad, San Diego, CA, USA). To compare fibre size and values of densitometric scanning of immune reactive bands of myosin protein normalized to actin between the control and peritonitis group, unpaired Student’s *t* test was used. A *P* value of <0.05 was considered statistically significant. Data are given as mean ± SD.

There is a known significant dependency of MRRP measurements on body muscle temperature, which varied from 36.3 to 39.8°C in the control pigs and from 33.8°C to 39.7°C in the peritonitis group [23]. Because of this sensitivity of MRRP, all the MRRP values were corrected to the mean control temperature of 37.9°C before making statistical comparisons, using the relationship for the 40 control recordings (MRRP = 12.0 - 0.253.T; Pearson *R* = −0.54, *P* = 0.00057)). In contrast to the high sensitivity of MRRP to temperature, ESN is relatively insensitive to temperature changes (*R* = 0.225, *P* = 0.17) as found previously [23].

## Results

**Figure 2:**
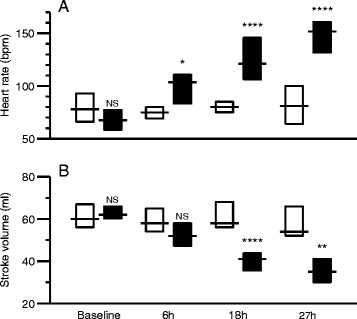
**Differences between control animals (white) and animals with peritonitis (black) at baseline, 6 hours, 18 hours and 27 hours after induction of sepsis. (A)** Heart rate, **(B)** stroke volume. Boxes indicate interquartile ranges and lines indicate median values. Comparisons between control and peritonitis animals were made with Welch rank test (NS = *P* >0.05, ^*^ = *P* <0.05, ^**^ = *P* <0.01, ^****^ = *P* <0.0001).

Characteristics of controls and animals with peritonitis at baseline and 27 hours thereafter, including weight, core temperature, systemic haemodynamics and laboratory measurements are summarized in Table 1. In septic pigs, there was a progressive increase of heart rate and decrease of stroke volume, whereas these parameters remained unchanged in controls over time (Figure 2). Septic pigs had lower blood pressure and markedly increased TNFα, higher lactate and lower pH and bicarbonate as compared to controls. Septic pigs received more fluids (7,150 ± 770 ml compared to 630 ± 550 ml in controls, *P* <0.01). Seven of ten peritonitic animals but none of the controls received norepinephrine to support the haemodynamics (*P* = 0.0031).

**Table 1:** **Laboratory characteristics of control animals and animals with peritonitis (values are given as mean ± SD)**

	**Controls (n = 10)**		**Wilcoxon**	**Peritonitis (n = 10)**		**Wilcoxon**	**Welch rank**
	**Baseline**	**27 hours**	**(** ***P*** **value)**	**Baseline**	**27 hours**	**(** ***P*** **value)**	**(** ***P*** **value)**
Weight (kg)	39.1 ± 3.7			40.2 ± 2.3			
Core temperature (°C)	38.50 ± 0.65	40.59 ± 0.34	0.0039^**^	38.16 ± 0.76	40.77 ± 0.37	0.002^**^	0.39
Mean arterial pressure (mmHg)	76.0 ± 14.3	88.6 ± 12.7	0.002^**^	78.6 ± 12.5	66.5 ± 19.9	0.16	0.009^**^
Heart rate (bpm)	80.1 ± 16.6	82.9 ± 20.7	0.82	68.5 ± 12.2	143.9 ± 25.4	0.002^**^	0.00009^****^
Cardiac output (l/min)	5.0 ± 1.3	4.6 ± 0.6	0.50	4.5 ± 0.9	4.8 ± 1.2	0.84	0.92
Stroke volume	63.2 ± 9.3	57.7 ± 10.1	0.65	65.9 ± 7.1	39.1 ± 13.7	0.004^**^	0.0011^**^
Sodium (mmol/l)	135.5 ± 1.9	141.5 ± 2.4	0.004^**^	134.3 ± 2.5	131.4 ± 2.6	0.19	0.00008^****^
Potassium (mmol/l)	4.0 ± 0.4	4.1 ± 0.2	0.61	4.0 ± 0.2	4.1 ± 0.4	0.76	0.76
Glucose (mmol/l)	8.4 ± 1.3	5.4 ± 0.4	0.002^**^	9.4 ± 1.3	5.0 ± 1.0	0.002^**^	0.21
Lactate (mmol/l)	1.2 ± 1.1	1.1 ± 0.4	0.77	1.0 ± 0.6	1.8 ± 0.7	0.05^*^	0.016^*^
pH	7.52 ± 0.04	7.47 ± 0.02	0.002^**^	7.51 ± 0.04	7.41 ± 0.06	0.004^**^	0.010^*^
Bicarbonate (mmol/l)	32.6 ± 1.8	31.4 ± 3.0	0.27	32.2 ± 1.5	26.9 ± 2.8	0.004^**^	0.004^**^
Base excess (mmol/l)	9.1 ± 2.7	7.9 ± 2.8	0.39	9.3 ± 1.9	2.5 ± 3.6	0.006^**^	0.006^**^
TNFα (pg/ml)	86.6 ± 76.2	60.9 ± 22.7	0.30	69.6 ± 46.9	317.6 ± 187.8	0.006^**^	0.002^**^

Wilcoxon test was used for comparison of baseline and 27-hour measurements within each group. Welch test of ranked data was used to compare 27-hour measurements between controls and animals with peritonitis. (^*^ = *P* <0.05, ^**^ = *P* <0.01, ^****^ = *P* <0.0001). TNFα, tumour necrosis factor alpha.

### Conventional electrophysiological investigations

**Figure 3:**
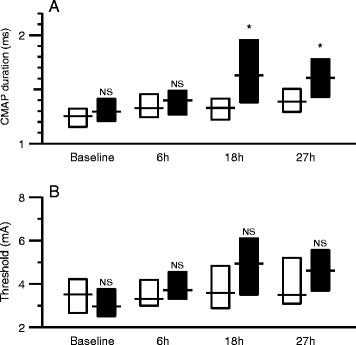
**Differences between control animals (white) and animals with peritonitis (black) at baseline, 6 hours, 18 hours and 27 hours after induction of sepsis. (A)** CMAP duration, **(B)** stimulus intensity to evoke an amplitude of 50% maximal CMAP. Boxes indicate interquartile ranges and lines indicate median values. Comparisons between control and peritonitis animals were made with Welch rank test (NS = *P* >0.05, ^*^ = *P* <0.05). CMAP, compound muscle action potential.

Results of motor nerve conduction studies are shown in Table 2 and Figure 3. In the peritonitis group, there was a significant increase of CMAP duration and stimulus intensity for 50% maximal CMAP at 27 hours, whereas both parameters remained unchanged in controls. None of the animals showed abnormalities (fibrillation potentials or positive sharp waves) in needle electromyography.

**Table 2:** **Nerve and muscle properties of controls and pigs with peritonitis (values are given as mean ± SD)**

	**Controls (n = 10)**		**Wilcoxon**	**Peritonitis (n = 10)**		**Wilcoxon**	**Welch rank**
	**Baseline**	**27 hours**	**(** ***P*** **value)**	**Baseline**	**27 hours**	**(** ***P*** **value)**	**(** ***P*** **value)**
***Nerve conduction studies***							
CMAP amplitude (mV)	1.62 ± 1.22	1.55 ± 1.08	0.19	1.05 ± 0.94	1.20 ± 1.17	0.19	0.80
CMAP duration (ms)	1.25 ± 0.11	1.40 ± 0.18	0.064	1.33 ± 0.14	1.61 ± 0.27	0.020^*^	0.044^*^
Stimulus for 50% maximal CMAP (mA)	3.74 ± 1.92	4.16 ± 2.04	0.77	3.32 ± 1.12	4.79 ± 1.51	0.0020^**^	0.35
***Muscle velocity recovery cycles***							
Muscle relative refractory period (ms)	2.41 ± 0.42	2.44 ± 0.46	0.63	2.38 ± 0.18	3.47 ± 1.79	0.0098^**^	0.0048^**^
Early supernormality (%)	8.28 ± 2.69	8.64 ± 1.96	0.63	9.64 ± 2.82	6.50 ± 2.64	0.0039^**^	0.0061^**^

(^*^ = *P* <0.05, ^**^ = *P* <0.01). CMAP, compound muscle action potential.

### Muscle velocity recovery cycles

**Figure 4:**
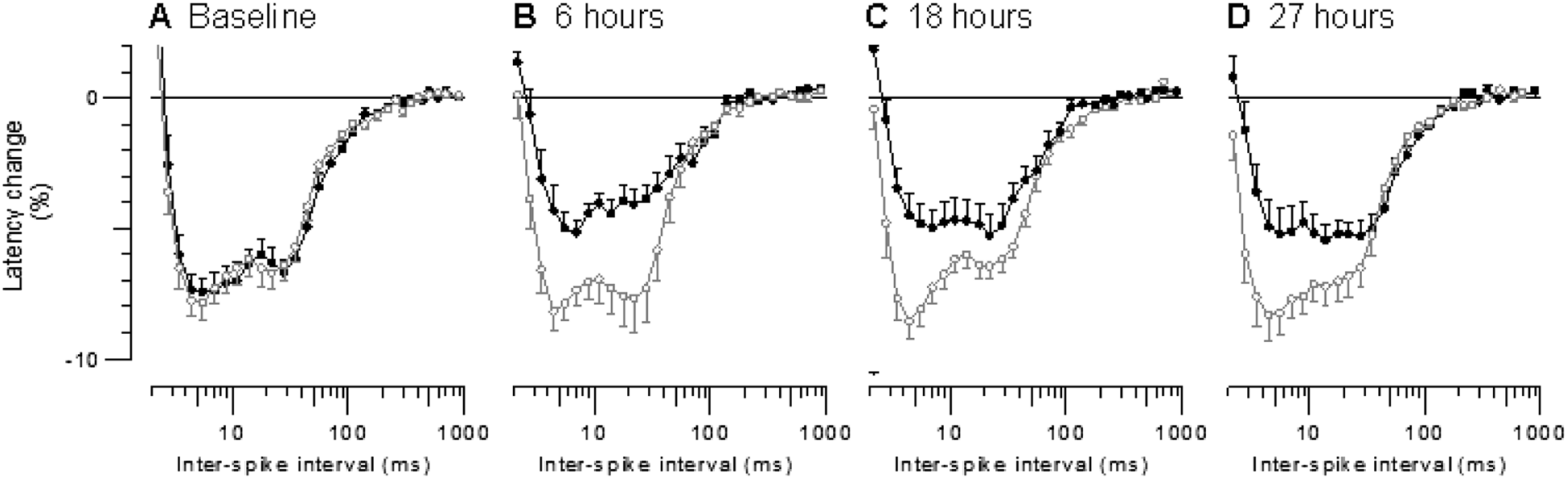
**Mean muscle velocity recovery cycles for the 10 control animals (open grey circles) compared with those for the 10 animals with peritonitis (filled black circles) at (A) baseline, (B) 6 hours, (C) 18 hours and (D) 27 hours after peritonitis induction.** Error bars indicate SEM.

Mean muscle VRCs for controls and animals with peritonitis are shown in Figure 4. Before peritonitis induction, mean muscle VRCs of both groups overlapped. After induction of peritonitis, VRCs of animals with peritonitis were shifted progressively to the right and upwards. In contrast, VRCs of controls did not change over time.

**Figure 5:**
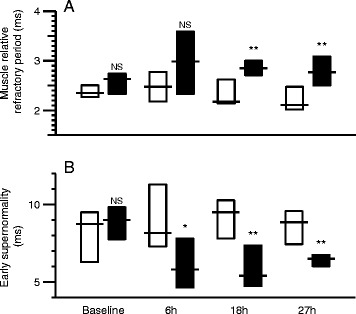
**Differences between control animals (white) and animals with peritonitis (black) at baseline, 6 hours, 18 hours and 27 hours after induction of sepsis. (A)** Muscle relative refractory period, **(B)** early supernormality. Boxes indicate interquartile ranges and lines indicate median values. Comparisons between control and peritonitis animals were made with Welch rank test (NS = *P* >0.05, ^*^ = *P* <0.05, ^**^ = *P* <0.01).

The shift to the right corresponds to a significant increase in MRRP in the peritonitis group, and a significant difference compared to the control group at 27 hours (Figure 5A, Table 2). Similarly, the shift upwards corresponds to a significant reduction in ESN in the peritonitis group, and a significant difference compared to the controls at 27 hours (Figure 5B, Table 2).

The differences between the control and peritonitis groups in MRRP and ESN developed early (Figure 5). Whereas baseline values of MRRP did not differ between groups, MRRP became progressively longer in the peritonitis group and was significantly different compared to controls 18 and 27 hours after induction of peritonitis (*P* ≤0.006) (Figure 5A).

ESN also did not differ between the groups at baseline, but was significantly reduced at 6, 18 and 27 hours (*P* ≤0.027) (Figure 5B).

### Muscle morphology and densitometric scanning of immune reactive bands of myosin normalized to actin

Mean cross-sectional area of skeletal muscle fibres was 1,490 ± 320 μm^2^ in the control and 1,450 ± 160 μm^2^ in the peritonitis group (*P* >0.05). Mean values of densitometric scanning of immune reactive bands of myosin normalized to actin were 0.26 ± 0.27 in the control and 0.13 ± 0.09 in the peritonitis group. This difference was not statistically significant (*P* >0.05). An additional file shows a representative skeletal muscle Western blot analysis and differences in densitometric scanning of immune reactive bands of myosin protein normalized to actin between control animals and animals with peritonitis (see Additional file 1).

## Discussion

Various studies have shown that muscle excitability measurements allow *in vivo* assessment of muscle membrane properties [15-19]. We have previously used this technique in critically ill patients diagnosed with probable CIM [20]. In the current study, muscle membrane excitability measurements were assessed in the early course of experimental peritonitis-induced sepsis in a pig model. The latter might be more suitable for studying sepsis-associated changes than rodent models [24]. Our results show that muscle membrane dysfunction occurred as early as 6 hours after induction of faecal peritonitis in pigs. There was a progressive reduction of ESN and prolongation of MRRP in the peritonitis group over 27 hours compared to controls. In parallel, conventional electrophysiology revealed an increased duration of CMAPs and an increase of stimulus threshold in peritonitis animals 27 hours after peritonitis induction.

Similar changes of muscle membrane properties have already been shown in critically ill patients diagnosed with probable CIM [20], during ischaemia [15,17] and in patients with chronic renal failure [16]. In probable CIM, we found a significant relationship between membrane dysfunction (that is fall in ESN and rise in MRRP) and potassium. Compared to patients with chronic renal failure, this relationship was much steeper in patients with probable CIM, suggesting that additional factors besides the expected dependence of membrane properties on potassium were involved [16,20]. The changes in our earlier studies during ischaemia were interpreted as a result of membrane depolarization, which was assumed to occur in ischaemia following ATP depletion and, therefore, failing of the Na^+^/K^+^ pump [15]. Ischaemia or tissue hypoxia due to inadequate tissue perfusion is likely to be also involved in sepsis. Furthermore, the changes in ESN and MRRP do not necessarily indicate membrane depolarization alone, since they can also occur because of increased sodium channel inactivation or reduced sodium channel availability [20]. In fact, earlier studies have demonstrated a selective inactivation of muscle sodium (Na_v_1.4) channels in CIM and reduced channel availability in a potential-dependent fashion [25-27].

Currently, the pathophysiology of sepsis-associated multiple organ failure, of which sepsis-induced myopathy and CIM may represent muscle manifestations, remains a matter of debate, and several mechanisms may be involved. Cell membrane potential changes have been observed in several experimental models, and proposed to be a pivotal mechanism for sepsis-related cellular dysfunction [28-32]. The evolution of cellular membrane potential changes during clinical sepsis has not been studied. Our results confirm the earlier reports on early muscle membrane potential changes in ICU patients obtained with invasive measurements [12]. Although, at the time the aforementioned study was carried out the entity of CIM was not yet described for the first time, the enrolled patient cohort very likely suffered with CIM. To the best of our knowledge, now no other study invasively measured muscle membrane potential in ICU patients because of the potential risks. Muscle VRC measurements provide a clinically feasible approach to study changes in muscle cell membrane properties non-invasively. As already mentioned, there is evidence that the toxic action of cytokines, anoxia or disturbances in the electrolyte gradients influence the resting membrane potential, provoking muscle membrane dysfunction [1,3,6]. Altered mitochondrial function has been proposed to contribute [33]. Nevertheless, in our animal model, faecal peritonitis had no measurable effects on skeletal muscle mitochondrial respiration [21]. All of the above-mentioned mechanisms could contribute to membrane dysfunction early in the course of sepsis-induced myopathy and/or CIM. Membrane dysfunction may act as the common mechanism for structural changes [5,6,8,9] in the course of disease.

Our study has some limitations. First, it was not possible to blind the investigators with respect to the different experimental groups. This is unlikely to cause bias, since we used well-defined treatment and recording protocols for all animals. Second, amount of fluid and therefore also sodium, as well as norepinephrine administration was higher and plasma sodium lower in the peritonitis group. We found no association between plasma sodium and excitability measurements in this study and earlier studies using the same methods [20]. We therefore believe that these differences are unlikely to explain the observed changes in sepsis. In a recent clinical observational study, the required amount of catecholamine was found to be a risk factor for the development of impaired direct muscle stimulation in a univariate but not in a multivariate Cox regression analysis [34]. We can relate our findings to sepsis-induced myopathy but not to CIM, since the observation period was too short and we did not find the morphometric changes of muscle fibres characteristic of CIM, and also there was no significant change of mean values of densitometric scanning of immune reactive bands of myosin normalized to actin. These findings are in line with reported data of studies using other animal models for CIM over a longer observational period if the findings within the first day are compared [35,36]. Another limitation is the use of two different mechanical ventilation modes. The pigs were randomly allocated in the mode of ventilation, and we studied peripheral muscles, not respiratory muscles, and found no differences in excitability parameters depending on the ventilatory mode. Overall, application of results from experimental sepsis in animal models to clinical sepsis must be done with caution.

## Conclusions

In summary, it seems likely that changes in muscle membrane properties provide an early indication of the development of CIM, although the underlying mechanisms are not completely understood. Muscle VRCs are a practicable tool for monitoring muscle membrane changes and may lead to earlier diagnosis of CIM in the future, and can facilitate further studies investigating the pathophysiology and prevention of this disease.

## Key messages

- Muscle VRCs can be used to assess changes of muscle membrane properties.
- In CIM, the muscle relative refractory period is prolonged and early supernormality reduced.
- Changes to muscle membrane properties occur early in the course of sepsis.

## Additional file

Additional file 1:
**Densitometric scanning of immune reactive bands of myosin normalized to actin.** Figure of representative skeletal muscle Western blot analysis and differences in densitometric scanning of immune reactive bands of myosin protein normalized to actin between control animals and animals with peritonitis.

## Abbreviations

CIM: critical illness myopathy
CIP: critical illness polyneuropathy
CMAP: compound muscle action potential
ESN: early supernormality
ICU: intensive care unit
MAP: mean arterial blood pressure
MRRP: muscle relative refractory period
SvO_2_: mixed venous oxygen saturation
TNFα: tumour necrosis factor alpha
VRC: velocity recovery cycle
