# Supplementary material for: Early changes of muscle membrane properties in porcine faecal peritonitis
Source: Crit Care. 2014 Aug 22;18(4):484. doi: 10.1186/s13054-014-0484-2 (PMC4159512; doi:10.1186/s13054-014-0484-2)
Supplement: Additional file 1: — Densitometric scanning of immune reactive bands of myosin normalized to actin. Figure of representative skeletal muscle Western blot analysis and differences in densitometric scanning of immune reactive bands of myosin protein normalized to actin between control animals and animals with peritonitis. [file 13054_2014_484_MOESM1_ESM.pdf]

A)

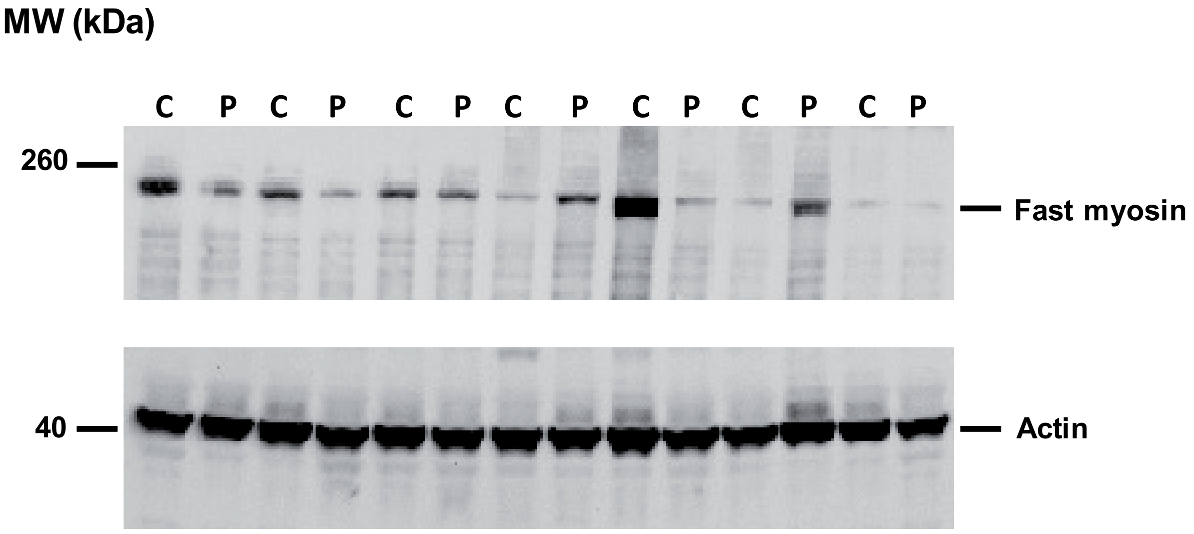

B)

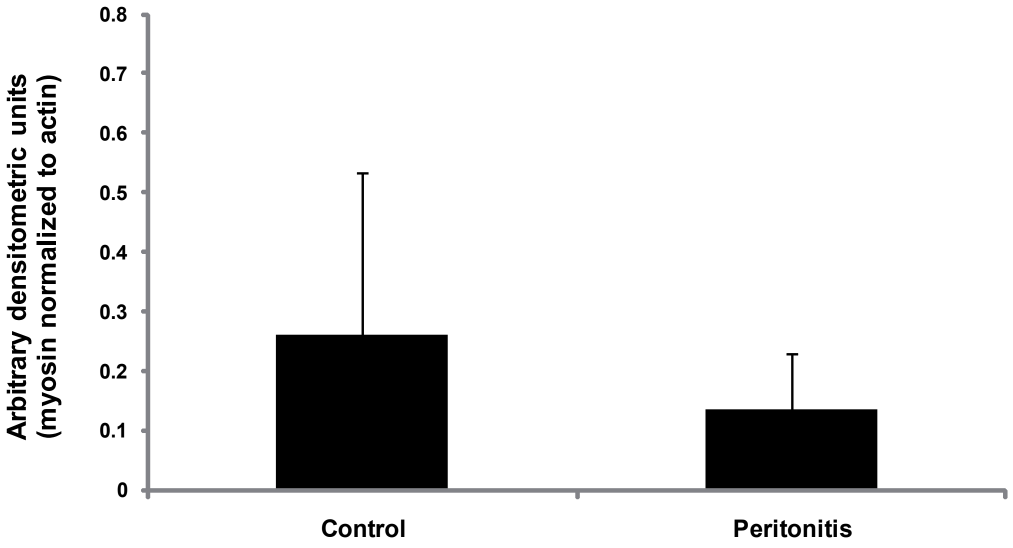

Figure legend:

(A) Representative skeletal muscle Western blot analysis. (C = control, P = peritonitis induced sepsis).

(B) Differences in densitometric scanning of immune reactive bands of myosin protein normalized to actin between control animals and animals with peritonitis (mean ± SD, unpaired Student's *t*-test $p = 0.19$).
